# Sexual Minority Adults and Smoking Cessation Outcomes: A Longitudinal Population-Based Study

**DOI:** 10.3390/healthcare14060705

**Published:** 2026-03-10

**Authors:** Steven A. Branstetter, Maya P. Matlack

**Affiliations:** Department of Biobehavioral Health, The Pennsylvania State University, University Park, PA 16802, USA; mpm7465@psu.edu

**Keywords:** smoking cessation, sexual minorities (SM), health disparities, epidemiology, cigarette smoking, longitudinal data

## Abstract

**Highlights:**

**What are the main findings?**

- Smoking cessation disparities were structured by the intersection of race, sex, and sexual orientation rather than by sexual minority status alone.
- Cessation outcomes differed across race, sex, and sexual orientation groups, with White heterosexual men showing the most favorable cessation profile and several other groups demonstrating a greater likelihood of continued smoking.

**What are the implications of the main findings?**

- Smoking cessation inequities reflect both behavioral dependence and broader structural disadvantages. Interventions may be more effective when they address smoking intensity and nicotine dependence alongside mental health burden and socioeconomic barriers, while also recognizing that risk is patterned by the combined influence of race, sex, and sexual orientation.
- Findings underscore the importance of tailoring cessation interventions for subgroups experiencing intersecting sexual and racial minority disadvantages.

**Abstract:**

**Background/Objectives:** Sexual minority (SM) adults experience higher cigarette smoking prevalence and poorer cessation outcomes than heterosexual adults, yet few empirically supported cessation programs are tailored to SM populations. In addition, the social and behavioral determinants of smoking disparities among SM remain understudied. A clearer understanding of factors associated with cessation in this population is necessary to inform targeted interventions. This study examined predictors of smoking cessation over a one-year period among sexual minority adults. **Methods:** Data were drawn from Waves 6 and 7 of the Population Assessment of Tobacco and Health (PATH) Study. Complex Samples logistic regression models assessed whether baseline smoking intensity, nicotine dependence, race, income, education, psychological distress, and quality of life predicted cessation one year later. An eight-category intersectional variable combined race, sex, and sexual orientation. **Results:** Smoking intensity and psychological distress were among the strongest predictors of cessation outcomes. Lower income and non-White race were also associated with reduced cessation likelihood. Cessation outcomes varied significantly across the combined race by sex by sexual orientation groups, with White heterosexual men exhibiting the most favorable cessation profile. Several other groups demonstrated reduced likelihood of quitting after accounting for smoking intensity, nicotine dependence, socioeconomic factors, psychological distress, and quality of life. **Conclusions:** Smoking cessation disparities operate across intersecting social identities. Behavioral dependence, socioeconomic disadvantages, and psychological distress collectively shape cessation outcomes. Effective interventions should address nicotine dependence within the broader structural and mental health contexts influencing racially and sexually minoritized populations.

## 1. Introduction

Although smoking rates in the United States are at the lowest levels since the 1965 Surgeon General’s report, nearly 480,000 Americans die every year due to cigarette smoking, and 16 million suffer from smoking-related illnesses [1]. Despite a nearly 10% reduction in smoking rates since 2009, significant disparities remain, including among those living in rural areas, persons of color, and sexual and gender minorities [2,3,4]. For example, compared to their heterosexual peers, sexual and gender minorities (SGM), including those who identify as lesbian, gay, bisexual, transgender, or queer, are significantly more likely to be cigarette smokers, continue smoking after a health crisis, be less successful at quitting, and are less likely to respond to generalized intervention messages [4,5,6,7].

Despite these significant disparities in smoking prevalence and negative outcomes, there are few empirically supported cessation programs specifically tailored to the needs of SGM populations [8,9]. Whereas there has been some success in improving the acceptability of tailored programs, few have resulted in improved cessation, reduced nicotine dependence, or an overall readiness to quit smoking [10,11]. Additionally, the understanding of social determinants of smoking among SGM populations lags behind that of both the majority and other minoritized populations [12]. Gaining a comprehensive understanding of the environmental and behavioral factors uniquely associated with smoking-related disparities in SGM populations is critical for developing tailored intervention approaches.

The minority stress model has been used as a theoretical framework to further the understanding of a range of health-related outcomes in minoritized populations, including cardiovascular risk, substance use and dependence, and depression and anxiety [13,14]. More recently, the minority stress model has been applied to SGM populations to help understand issues such as harmful use of alcohol and cannabis, substance use disorders, and other mental health issues such as depression, anxiety, and suicidality [15]. Indeed, the minority stress model would suggest that SGM populations face unique challenges when attempting to quit smoking, including elevated chronic stress due to discrimination, rejection, harassment, internalized stigma, healthcare discrimination, and economic and structural inequity [16,17]. These issues may be further compounded by gender, with sexual minority men and women each facing distinct barriers to cessation [18]. Race also needs to be considered when examining the experiences of SGMs, as SGMs of color may experience intersecting discrimination based on their race and SGM status [19,20]. There is also discrimination even within SGM groups. Research has shown that groups may discriminate against other SGM groups, such as transgender or bisexual individuals [21,22]. However, research is lacking in terms of other risk and protective factors that may influence the impact of minority stress on smoking cessation. For example, some factors (such as low SES) may compound with minority stress to produce worse smoking outcomes; other factors (such as high SES) may serve as buffers that reduce the negative impact of minority stress among SGMs.

In order to apply the intersectional minority stress framework to smoking cessation, the current paper seeks to: (1) identify key behavioral and socio-demographic factors associated with smoking cessation over a one-year period in an adult SGM population, (2) examine how these factors differ by SGM groups, and (3) examine how they interact with sex. Specifically, we examined how nicotine dependence, smoking frequency, race, income, education, employment, and mental distress at baseline were associated with successful cessation at one-year follow-up. Drawing on the minority stress framework and intersectionality theory, we hypothesized that (1) sexual minority smokers would demonstrate lower likelihood of quitting relative to heterosexual smokers after adjustment for behavioral and socioeconomic factors; (2) racially minoritized smokers would exhibit lower cessation likelihood relative to White smokers; and (3) disparities would be most pronounced among individuals with both racial and sexual minority identities. We further hypothesized that psychological distress and socioeconomic disadvantages would partially account for observed disparities. Findings may inform tailored smoking cessation intervention approaches that address key behavioral and social determinants associated with quitting among a population experiencing minority stress.

## 2. Materials and Methods

### 2.1. Study Design and Participants

Data for the present study are drawn from Wave 6 (baseline) and Wave 7 (follow-up) of the Population Assessment of Tobacco and Health (PATH) study [23]. The PATH study is a nationally representative, longitudinal cohort study of tobacco and nicotine use among youth and adult populations living in the United States beginning in 2013. Wave 6 data were collected in 2021, and Wave 7 data were collected in 2022–2023. Full details on PATH methodology, sampling, and data collection are available in Hyland and colleagues (2017) [23] and through the U.S Department of Health and Human Services [24].

The analytic sample for the present study comprised 4449 adult participants aged 18 years or older who reported cigarette smoking at Wave 6 and who also participated in Wave 7 data collection. All analyses were conducted using the appropriate PATH longitudinal sampling weights and Complex Samples procedures to ensure nationally representative estimates and design-corrected variance estimation.

### 2.2. Measures

#### 2.2.1. Demographics and Socioeconomic Factors

Age. Participant’s age at Wave 6 was reflected in one of six categories: (1) 18–24, (2) 25–34, (3) 35–44, (4) 45–54, (5) 55–64, and (6) 65+.

Race/Ethnicity. For the purposes of this study, race was categorized into three groups: (1) White alone, (2) Black alone, and (3) Other.

Educational Attainment. Education was grouped as: (1) less than high school, (2) GED, (3) high school graduate, (4) some college or associate’s degree, and (5) bachelor’s or advanced degree.

Household Income. Annual household income was categorized into five levels: (1) less than $10,000, (2) $10,000–$24,999, (3) $25,000–$49,999, (4) $50,000–$99,999, and (5) $100,000 or more.

Employment Status. Current employment status was categorized as: (1) full-time (35+ hours/week), (2) part-time (15–34 h/week), (3) part-time (<15 h/week), and (4) not currently working.

Sexual Identity. In addition to measuring sexual identity as 1 = straight, 2 = lesbian or gay, and 3 = bisexual, and self-reported sex as 1 = male and 2 = female, a four-level variable was created to identify sexual minority status by sex, resulting in the following categories: 1 = male sexual minority, 2 = female sexual minority, 3 = male heterosexual, and 4 = female heterosexual. For analyses, this variable was dummy coded with male heterosexuals serving as the reference group.

#### 2.2.2. Smoking

Smoking Frequency. The frequency of smoking cigarettes was measured as the self-reported average number of cigarettes smoked per day in the last 30 days.

Nicotine Addiction. Addiction to nicotine was measured as the time to first cigarette after waking. Time to first cigarette is a well-established and robust single-item indicator of nicotine dependence, shown to be predictive of both physiological dependence and cessation outcomes [25,26,27].

Smoking Cessation. Participants who reported current smoking at Wave 6 and indicated no past 30-day cigarette use at Wave 7 were classified as having quit smoking.

#### 2.2.3. Mental Health/Well-Being

Depression/Mental Distress. The item “Last time you had significant problems with feeling very trapped, lonely, sad, blue, depressed, or hopeless about the future” was used as a proxy measure for general depressive symptoms and mental distress. Participants responded to the question on a 4-point ordinal scale: (1) past month, (2) 2–12 months ago, (3) more than a year ago, and (4) never.

Quality of life. Participants rated their overall quality of life on a 5-point scale: (1) excellent, (2) very good, (3) good, (4) fair, and (5) poor.

### 2.3. Analytic Approach

All analyses were conducted using IBM SPSS (version 31) Statistics Complex Samples procedures (IBM Corp., Armonk, New York, NY, USA, 2023). The binary outcome variable was smoking cessation from Wave 6 to Wave 7 (quit/did not quit). To account for the complex sampling design of the PATH Study, all regression analyses were performed using Taylor series linearization with the Wave 7 longitudinal adult weight, incorporating stratification and clustering variables. This approach produced nationally representative estimates and design-corrected standard errors.

For primary analyses, a fully adjusted Complex Samples logistic regression model was conducted with predictors including sex and sexual orientation, race, household income, educational attainment, psychological distress, quality of life, cigarettes per day at Wave 6, and time to first cigarette. Odds ratios and 95% confidence intervals were derived from the design-based parameter estimates, and overall model significance was evaluated using Wald F statistics rather than likelihood ratio chi-square tests, consistent with complex survey estimation procedures.

To examine whether cessation disparities reflected the combined and potentially compounding effects of race, sex, and sexual identity, an intersectional Complex Samples logistic regression model was conducted. Participants were classified into one of eight mutually exclusive groups defined by sex (male, female), sexual identity (heterosexual, sexual minority), and race (White, non-White). Statistical significance of predictors was assessed using design-based Wald F tests. Effect estimates were summarized as odds ratios with 95% confidence intervals derived from complex survey-adjusted parameter estimates.

## 3. Results

### 3.1. Sample Characteristics

The analytic sample included 4449 adult smokers with valid Wave 6 to Wave 7 follow-up data. In the unweighted sample, 42.1% identified as heterosexual men (n = 1846), 43.5% as heterosexual women (n = 1910), 10.2% as sexual minority women (n = 446), and 4.3% as sexual minority men (n = 188). Using the Wave 7 longitudinal weight and design-based variance estimation, heterosexual men comprised 49.6% of the population, heterosexual women 39.0%, sexual minority women 7.5%, and sexual minority men 3.9%. The weighted racial composition was 77.0% White and 23.0% non-White. Overall, the weighted quit rate from Wave 6 to Wave 7 was 13.3%. Additional baseline demographic information is presented in Table 1.

### 3.2. Regression Analyses

The overall adjusted Complex Samples logistic regression model was statistically significant, Wald F(20, 80) = 12.26, *p* < 0.001. Pseudo R^2^ statistics indicated modest explanatory power (Cox & Snell R^2^ = 0.088, Nagelkerke R^2^ = 0.165, McFadden R^2^ = 0.121).

#### 3.2.1. Main Effects of SM on Cessation

Sex and sexual orientation were significantly associated with smoking cessation in the fully adjusted Complex Samples logistic regression model, Wald F(3, 97) = 3.21, *p* = 0.026. Compared to heterosexual men, heterosexual women demonstrated significantly higher odds of quitting smoking, OR = 1.32, 95% CI [1.08, 1.61], *p* = 0.008. In contrast, sexual minority men did not significantly differ from heterosexual men in the likelihood of cessation after adjustment for smoking behavior, socioeconomic position, race, and mental health indicators, OR = 0.91, 95% CI [0.63, 1.31], *p* = 0.602. Similarly, sexual minority women did not significantly differ from heterosexual men in the fully adjusted model, OR = 1.07, 95% CI [0.84, 1.36], *p* = 0.586

#### 3.2.2. Demographic, Behavioral, and Socioeconomic Predictors

Smoking behavior was the strongest predictor of cessation outcomes in the adjusted model. Each additional cigarette per day at Wave 6 was associated with substantially lower odds of quitting at Wave 7, OR = 0.903, 95% CI [0.880, 0.926], Wald F(1, 98) = 63.593, *p* < 0.001. Time to first cigarette also contributed independently, Wald F(1, 98) = 5.490, *p* = 0.021, but the magnitude of this association was comparatively small once cigarettes per day was included (Exp[B] = 1.001 per unit increase in time to first cigarette).

Race and socioeconomic position were also associated with cessation. Race was significant in the fully adjusted model, Wald F(1, 98) = 4.585, *p* = 0.035. White participants demonstrated higher odds of cessation relative to non-White participants, OR = 1.356, 95% CI [1.027, 1.790]. Likewise, household income was also significantly associated with outcomes, Wald F(4, 95) = 2.625, *p* = 0.039. Relative to the higher income, each lower income category had lower adjusted odds of cessation, with odds ratios ranging from OR = 0.589 to OR = 0. Educational attainment was not significant in the fully adjusted model, Wald F(4, 95) = 1.609, *p* = 0.178.

### 3.3. Intersectional Model

Mental health and well-being variables showed mixed evidence in the fully adjusted model. Psychological distress was significantly associated with cessation, Wald F(3, 96) = 5.419, *p* = 0.002, indicating that cessation likelihood varied across distress categories even after adjustment. Quality of life was not statistically significant in the fully adjusted model, Wald F(4, 95) = 1.887, *p* = 0.119. To examine whether cessation disparities were shaped by overlapping social identities, an intersectional variable combining race, sex, and sexual orientation was constructed, yielding eight mutually exclusive categories with White heterosexual men specified as the reference group. The fully adjusted Complex Samples logistic regression model incorporating this intersectional variable was statistically significant, Wald F(20, 79) = 9.714, *p* < 0.001, with pseudo R^2^ values suggesting modest explanatory power (Cox and Snell = 0.089; Nagelkerke = 0.166; McFadden = 0.122). The intersectional factor itself was statistically significant, Wald F(6, 93) = 3.005, *p* = 0.010, indicating meaningful heterogeneity in cessation outcomes across the combined race by sex by sexual orientation groups even after accounting for smoking intensity, nicotine dependence, income, education, psychological distress, and quality of life.

Because the model estimated the odds of quitting, odds ratios greater than 1.00 reflect an increased likelihood of smoking cessation relative to White heterosexual men. In the fully adjusted intersectional model, White heterosexual men exhibited the most favorable cessation outcomes. Non-White heterosexual men demonstrated lower odds of cessation compared to White heterosexual men, OR = 0.823, 95% CI [0.401, 1.691]. Likewise, White heterosexual women showed substantially lower odds of quitting, OR = 0.414, 95% CI [0.182, 0.942]. Finally, non-White heterosexual women also demonstrated reduced odds of quitting, OR = 0.487, 95% CI [0.236, 1.004]. Among sexual minority subgroups, White sexual minority men (OR = 0.453, 95% CI [0.200, 1.029]), non-White sexual minority men (OR = 0.709, 95% CI [0.250, 2.007]), White sexual minority women (OR = 0.694, 95% CI [0.231, 2.089]), and non-White sexual minority women (OR = 0.508, 95% CI [0.242, 1.063]) all showed odds ratios below 1.00 relative to White heterosexual men, indicating lower cessation likelihood in each group.

The overall statistical significance of the intersectional factor indicates that cessation disparities were structured by the combined configuration of race, sex, and sexual orientation rather than by any single identity dimension alone. Although sexual minority status did not uniformly predict lower cessation in the main-effects model once behavioral and socioeconomic covariates were included, disparities became clearer when identities were examined jointly. For full results, see Table 2. 

## 4. Discussion

The present study examined social and behavioral determinants of smoking cessation over a one-year period among adult smokers using nationally representative longitudinal data and appropriate design-based variance estimation. Results suggest that smoking intensity and psychological distress are among the strongest predictors of cessation outcomes. Socioeconomic disadvantages and race were also associated with reduced likelihood of quitting. Importantly, cessation disparities were structured in an intersectional manner, such that the configuration of race, sex, and sexual orientation shaped cessation profiles in patterned ways.

When sex and sexual orientation were examined in the fully adjusted model, heterosexual women demonstrated higher odds of quitting relative to heterosexual men, whereas sexual minority men and sexual minority women did not significantly differ from heterosexual men after accounting for smoking behavior, socioeconomic position, and mental health. These findings suggest that sexual minority status alone does not uniformly determine cessation outcomes. Rather, cessation appears to reflect the broader behavioral and structural context in which smoking occurs.

Heavier smoking at baseline substantially reduced the likelihood of quitting, reinforcing that smoking intensity remains a primary driver of cessation outcomes. Nicotine dependence, measured as time to first cigarette, demonstrated a statistically reliable but comparatively small independent association once smoking intensity was included. Together, these findings indicate that behavioral dependence continues to play a central role in cessation across identity groups.

Socioeconomic conditions also shaped cessation over the one-year follow-up. Lower household income was associated with reduced likelihood of quitting, independent of smoking behavior and mental health. Race was similarly associated with cessation, with non-White smokers demonstrating lower cessation likelihood relative to White smokers even after adjustment for income and education. These patterns suggest that racial disparities in cessation are not explained solely by socioeconomic position and may reflect broader structural and contextual influences.

Psychological distress emerged as one of the more robust predictors of reduced likelihood of quitting. Individuals reporting more recent distress were significantly less likely to quit, consistent with prior research linking mental health burden to smoking persistence and relapse. Smoking may function as a coping mechanism in the context of ongoing stress, and cessation efforts that do not address psychological distress directly may be less effective [28,29].

The intersectional analysis provided the clearest evidence of patterned disparities. When race, sex, and sexual orientation were combined into an eight-category variable, meaningful differences in cessation outcomes persisted across groups even after adjusting for smoking intensity, nicotine dependence, income, education, psychological distress, and quality of life. White heterosexual men exhibited the most favorable cessation profile. Several other groups, including non-White heterosexual men, non-White heterosexual women, White heterosexual women, and sexual minority subgroups, demonstrated an elevated likelihood of continued smoking relative to this reference group [30].

The largest disparities were observed among non-White heterosexual men and non-White heterosexual women, underscoring the continued role of race in structuring cessation outcomes. Sexual minority subgroups also exhibited an elevated likelihood of continued smoking, although these differences were generally smaller in magnitude than some racially minoritized heterosexual groups. Importantly, these differences remained after controlling for behavioral dependence and psychological distress, indicating that smoking intensity and mental health burden do not fully account for intersectional disparities.

These findings are consistent with intersectional and minority stress perspectives, which posit that health disparities emerge from overlapping systems of social stratification. Individuals located at the intersection of racial and sexual minority identities may experience layered stressors and structural constraints that extend beyond measurable socioeconomic indicators. At the same time, the heterogeneity observed across groups cautions against broad generalizations. Disparities were patterned by specific combinations of identities rather than by sexual minority status alone.

Quality of life was not independently associated with cessation in the fully adjusted model [31]. Although conceptually relevant, the single-item measure used here may not have captured the multidimensional aspects of well-being that influence smoking behavior. Future research incorporating more comprehensive measures may clarify how subjective well-being interacts with cessation across diverse identity groups.

Several limitations should be noted. Measures of mental distress and quality of life were brief and may not fully capture minority stress processes. The publicly available PATH measures of sex and sexual identity were limited in scope, including binary sex categories and constrained sexual identity response options, which limited the ability to examine gender-diverse populations. The study also did not assess reasons for quitting, quit attempts, or cessation methods. Smoking cessation was defined as the absence of past 30-day cigarette use at one-year follow-up, which captures real-world quitting patterns but does not distinguish between voluntary cessation, health-driven cessation, or other pathways. Additionally, although this definition of cessation is widely used in population-based epidemiologic research, it does not distinguish between sustained abstinence and short-term interruption. Consequently, the present findings reflect cessation status rather than cessation processes, limiting insight into mechanisms underlying observed disparities. Finally, although the minority stress framework guided the conceptual framing of this study, data did not allow for a comprehensive assessment or measurement of discrimination, internalized stigma, or healthcare-based mistreatment. Psychological distress and socioeconomic indicators may partially reflect exposure to chronic stressors, but they do not directly capture minority-specific stress processes. Future studies should incorporate more nuanced measures of gender identity, sexual orientation, minority stress, and cessation strategies to better understand mechanisms underlying observed disparities.

Taken together, the findings of this indicate that cessation disparities are not equally distributed across sexual minority populations but are structured by intersecting configurations of race, sex, and sexual identity. For healthcare systems and public health programs, this suggests that cessation interventions targeting ‘sexual minorities’ as a single category may overlook meaningful heterogeneity in risk and needs. These findings carry implications for tobacco control policy and healthcare delivery [32]. Moreover, policy-level strategies for community-based cessation programming and structural discrimination within healthcare settings may be necessary to meaningfully reduce smoking disparities.

## 5. Conclusions

Smoking cessation disparities are structured by the combined configuration of race, sex, and sexual orientation rather than by any single identity dimension alone. In the fully adjusted intersectional model, White heterosexual men exhibited the most favorable cessation profile. Several other groups, including non-White heterosexual men, non-White heterosexual women, White heterosexual women, and sexual minority subgroups, demonstrated reduced likelihood of quitting even after accounting for nicotine dependence, smoking intensity, income, education, and psychological distress.

Taken together, these findings highlight the multidimensional and compounding nature of smoking cessation inequities. Behavioral dependence, mental health burden, and socioeconomic disadvantage operate within broader social contexts shaped by intersecting identities. Efforts to reduce tobacco-related disparities must therefore extend beyond nicotine dependence and incorporate strategies that address mental health and structural barriers, particularly for racially and sexually minoritized subgroups, in order to promote more equitable cessation outcomes.

## Figures and Tables

**Table 1:** Baseline characteristics of the analytic sample and cessation rates from Wave 6 to Wave 7.

Group (Sex by Sexual Identity)	n	%	Quit Rate (Wave 6 to Wave 7), %
Heterosexual men	1846	50.0	14.0
Heterosexual women	1910	38.3	10.9
Sexual minority women	446	7.5	18.4
Sexual minority men	188	4.1	19.9
Total	4390	100.0	13.4

**Table 2:** Weighted intersectional logistic regression predicting smoking cessation from Wave 6 to Wave 7 among adult smokers.

Predictor	B	OR	Wald F (df1, df2)	*p*
Intersectional group (race x sex x sexual identity)			2.691 (7, 92)	0.014
Non-White sexual minority women	−0.194	0.823		
Non-White heterosexual men	0.687	1.987		
White heterosexual women	0.525	1.691		
Non-White heterosexual women	0.597	1.817		
White sexual minority men	0.149	1.161		
Non-White sexual minority men	0.171	1.186		
White sexual minority women	0.484	1.622		
Constant	1.531	4.622		

## Data Availability

The data presented in this study are openly available as the Population Assessment of Tobacco and Health (PATH) Study [United States] Public-Use Files through the Inter-university Consortium for Political and Social Research (ICPSR), DOI: 10.3886/ICPSR36498.v17.
